# Assessment of green lentil malt as a substrate for gluten-free beer brewing

**DOI:** 10.1038/s41598-023-50724-x

**Published:** 2024-01-04

**Authors:** Alan Gasiński, Joanna Kawa-Rygielska

**Affiliations:** Department of Fermentation and Cereals Technology, Faculty of Biotechnology and Food Sciences, Wrocław University of Environmental and Life Sciences, Chełmońskiego 37 Street, 51-630 Wrocław, Poland

**Keywords:** Agroecology, Nutrition

## Abstract

The aim of this study was to analyze whether it is possible to brew beer without using cereals so that the produced beverage could be easily accessible for the population suffering from celiac disease and other gluten-related disorders. Green lentil seeds were malted and then mashed using a congress mashing procedure to assess their advantages and disadvantages in the brewing process. Based on the congress mashing procedure, the mashing process needed to produce beer was developed, and beers were produced from the lentil malts germinated during malting for 96 h, 120 h and 144 h. It was possible to produce beers from the lentil malts; however, they were characterized by a lower alcohol content, lower degree of attenuation and some discrepancies between the concentrations of various volatiles (such as acetaldehyde, ethyl acetate, and 1-propanol) compared to the control beer produced from barley malt.

## Introduction

Lentil is a popularly grown high-protein legume that is drought resistant and can be grown in various climates, as it is able to thrive in warm and cool environments^1^. Lentil seeds are used in many cuisines to prepare a vast array of different dishes, which might also mean that various populations could be interested in novel lentil-based food products^2^. Beer, however, is a beverage not typically produced from lentils but from 4 main ingredients: water, malt, hops and yeast. Malting is a process that is used primarily to modify barley grains, and the main reason for malting is to increase the enzymatic activity of the modified seeds, primarily amylases^3^. On a smaller scale, malts are also produced from various cereals other than barley, such as wheat, rye, and oats, or from various pseudocereals, such as buckwheat. These malts are used in the production of various specialty beers, which are characterized by different organoleptic properties than the typical barley malt beer. Legumes are not used in beer brewing technology; however, in recent years, few trials on malting various legume seeds and analyzing the technological properties of these malts have been conducted. Gasiński et al.^4^ malted various legume seeds (such as lentil, common vetch, chickpea and peas) in conditions typical for the production of Pilsner-type barley malt, but the acquired malts were characterized by insufficient technological parameters for beer brewing: they had not saccharified during mashing, were filtered for a long time and wort produced from these malts possessed very low extract content. Even the addition of various commercial brewing enzyme preparations during mashing did not allow for the production of acceptable wort. In a subsequent study, where lentil steeping was performed under conditions that allowed lentil to acquire up to 65% water content and then germinate at a higher temperature than is typical for Pilsner-type barley malt, wort from green lentil malt prepared in this way was characterized by improved parameters compared to the last study^5^. However, lentil malts still had not fully saccharified during mashing, and acquired worts were characterized by suboptimal extract content. Data acquired in these two studies have shown that changing simple malting characteristics resulted in significantly improved properties of this malt type. Additionally, Trummer et al.^6^ showed that lentil malt can be used as an additive to beer grain bill in amounts of up to 20%. However, these data still do not explain why there is an interest in the production of lentil malt. Malts do not always have to be used in the production of wort for beer production but can function as a substrate for various other food products. Typically, malting changes the concentration of vitamins, improves the bioavailability of minerals, reduces the concentration of various antinutritional substances, improves the digestibility of carbohydrates and proteins and changes the organoleptic properties of malted seeds^5,7–9^. Additionally, due to the increasing temperatures in many of the world regions due to climate change, barley might not be a suitable brewing material in the future because the starch gelatinization temperature of cereals, barley included, increases when plants are cultivated in warm environments^10,11^. This might prove very problematic for brewers, which is why the assessment of alternative substrates for beer production is reasonable. Additionally, consumers are currently looking for new flavors and fragrances in beer, and as lentil is a rich source of proteins and yeast produces various flavor-active volatile components from the amino acids present in the wort, lentil malts could possibly be used as a means to produce novel-tasting beer styles^12,13^. Furthermore, various cereals and cereal-based products cannot be consumed by people suffering from celiac disease, nonceliac gluten sensitivity, dermatitis herpetiformis, gluten ataxia and other disorders, which are diagnosed in a far larger percent of the world’s population than in the years before^14,15^. In this study, green lentil seeds were malted in a malting procedure used sometimes to produce diastatic malts and then mashed with various adjuncts to determine whether simple adjustments to the composition of the mash can result in full saccharification of the starch of the lentil malt^9^. In the second part of the study, green lentil malt was used in a mashing regime designed for the parameters of this malt to produce novel lentil-based beers.

## Materials and methods

### Materials

#### Raw material

The plant materials used in this study were seeds of green lentil (*Lens culinaris*) of the Eston variety. Lentil seeds were acquired from BioPlanet company (Leszno near Warsaw, Poland). Lentil seeds, prior to the malting procedure and analyses, were manually sifted to discard damaged seeds and seeds with visible discoloration. The moisture content of the seeds before the malting process was analyzed with an MT moisture analyzer (Brabender, Duisburg, Germany). All techniques and analyses used on the raw material were carried out in accordance with relevant institutional, international and national guidelines and legislation.

#### Biological material

The biological material used in this study was S-04 *Saccharomyces cerevisiae* yeast (Fermentis, Paris, France), added to the wort in the amount recommended by the producer (0.5 g per 1dm^3^ of wort). Yeast (8.75 g) was rehydrated in 100 cm^3^ of sterile, distilled water at 20 °C 20 min prior to inoculation. All techniques and analyses used on the biological material were carried out in accordance with relevant institutional, international and national guidelines and legislation.

#### Reagents and standards

The standards used in this study were acetaldehyde (2,3-butanodione), ethyl acetate, 1-propanol, 2-butanol, 2-methylbutanol, 3-methylbutanol, ethyl hexanoate, ethyl octanoate, ethyl decanoate, furfural, isobutanol (2-methylpropan-1-ol), isopentyl acetate (isoamyl acetate) and phenylethyl alcohol (purity of the standards was equal to or higher than 99%, suitable for GC analyses) purchased from Merck (Merck KGaA, Darmstadt, Germany). Reagents used in this study were calcium carbonate (99%), sodium hypochlorite (15%), aqueous iodine (2% *w/v* of iodine), diatomaceous earth (Chempur, Piekary Śląskie, Poland) and α-amylase from *Bacillus* sp. solution (amylolytic activity equal to 28,345 units per cm^3^ of solution, where 1 unit will liberate 1 mg of maltose from starch in 3 min. at pH 6.9 at 20 °C) (Merck KGaA, Darmstadt, Germany).

### Methods

#### Malting procedure

Eighty grams of green lentil was weighed and transferred to perforated, stainless steel malting containers (total of 24 containers), which were previously disinfected by drying in a UF110 Plus dryer (Memmert GmbH + Co, Schwabach, Germany) for 2 h at 200 °C and then cooled to room temperature. Containers filled with a known mass of lentil were then weighed (the container filled with lentil seeds will be referred to as the ‘malting kit’). Changes in the moisture content of the seeds during the first step of the malting process (steeping) were calculated based on the changing weight of the malting kit, assuming that the increase in weight of the kit is equal to the quantity of water adsorbed by the seeds. Steeping was executed in the water‒air steeping cycle. At the start of the process, malting kits were submerged in 1.5% sodium hypochlorite solution for 10 min to surface sterilize the seeds. Malting kits were then removed from the sodium hypochlorite solution and washed three times with distilled water. After this process, malting kits were submerged in sterile tap water (disinfected previously by boiling and cooled) at a temperature of 15 °C for 7 h, transferred to the KK 240 Smart Pro germination chamber (with humidity set at 90% relative humidity and temperature set at 18 °C) for 17 h, submerged another time in fresh, disinfected tap water at a temperature of 15 °C for 4 h, and then transferred to the germination chamber (temp. 18 °C, relative humidity 90%) for 20 h. After each step, malting kits were weighed to determine the changing moisture content of the lentil seeds. At the end of the steeping process, the moisture content of the lentil seeds was equal to 62–62.5%. Lentils were germinated in the germination chamber with the relative humidity set at 90%. Lentils were divided into three different batches of eight containers, which were germinated for different amounts of time. One batch was germinated for 96 h, one batch for 120 h and one batch germinated for 144 h. The temperature during germination was programmed as follows: 18 °C for the first 24 h; 15 °C for the second 24 h and 12 °C for the remainder of the germination time (48 h, 72 h or 96 h, depending on the batch of malt produced). After the germination process, each batch of malting kits was dried in a UF110 Plus dryer at 50 °C for 23 h^4,5^. The malting procedure resulted in the production of 3 different lentil malt samples:

- Green lentil malt germinated for 96 h (4 days) (GR4)
- Green lentil malt germinated for 120 h (5 days) (GR5)
- Green lentil malt germinated 144 h (6 days) (GR6)

Malts of one type from different containers, after the drying process, were mixed together and transferred to tightly closed containers to prevent moisture absorption during the cooling period. Malts, as well as unmalted lentils, were ground with the use of a Bühler Miag disc mill DLFU (Bühler, Uzwil, Switzerland), according to the Analytica EBC 4.5.1 method for the subsequent analysis^16^.

#### Analysis of the technological properties of the malts on the basis of the congress mashing procedure.

Technological properties of the green lentil malts, such as saccharification time, wort filtration time, wort volume, wort extract and wort pH, were assessed on the basis of the Analytica EBC 4.5.1 congress mashing procedure^16^. Additional factors, such as the influence of calcium ions in the mash or the addition of α-amylase solution on the mashing performance, were also analysed. Mashing was performed in the twelve-cup automated mashing apparatus LB-12 (LB Electronic, Berching, Germany). The basic procedure used to analyze solely lentil malts was as follows: the mashing cup was filled with 200 cm^3^ of distilled water and heated to 45 °C. Fifty grams of ground malt was added to the mashing cups followed by stirring (100 rpm) of the mash. The 45 °C temperature was maintained for 30 min, after which the temperature of the water bath in the mashing machine was increased to 70 °C at a rate of 1 °C per min. One hundred cm^3^ of distilled water at 70 °C was added to the mashing cups. Ten minutes after the addition of 100 cm^3^ of water, the saccharification time measurement was started. Saccharification of the mashes was assessed via the iodine test in 5 min intervals. A temperature of 70 °C was maintained for 60 min, after which the contents of the mashing cups were cooled to 20 °C. The mash weight was adjusted to 450 g using distilled water. Mashes were transferred to laboratory funnels (20 cm in diameter) fitted with Macherey–Nagel MN 614 ¼ filters (320 mm in diameter) (Macherey–Nagel GmbH & Co, Düren, Germany). To rinse the remaining malt, 100 cm^3^ of the filtered wort was reversed to the mashing cup and poured back into the funnel. After reversing the first 100 cm^3^ of wort, the filtering of the congress worts lasted up to 120 min. After filtration, wort was collected for analyses. The worts were prepared in duplicate. The volume of the wort was read from the scale of the cylinder. The extracted content of the worts, cooled to 20 °C, was assessed using a DMA 35 densimeter (Anton Paar, Graz Austria) in triplicate. Wort pH was assessed using an MP220 pH meter (Mettler Toledo, Columbus, OH, USA) in triplicate for each wort sample^4,16,17^.

To analyze the influence of the calcium ions on the mashing performance, 100 cm^3^ of distilled water in the mashing cup (at the temp. of 45 °C) was substituted by 100 cm^3^ calcium carbonate solution (with the Ca^2+^ ion concentration equal to 50 mg/dm^3^). To analyze the influence of alpha-amylase solution, 0.01 cm^3^ of alpha-amylase solution was added to the mash at 70 °C after the addition of 100 cm^3^ of water. This mashing regime allowed the analysis of the mashing properties of the lentil malts in 9 different variants:

- Lentil malt germinated for 96 h and mashed under a congress mashing regime (4D)
- Lentil malt germinated for 96 h and mashed under a congress mashing regime (4D-Ca) with the addition of calcium ions
- Lentil malt germinated for 96 h and mashed under a congress mashing regime (4D-Am) with the addition of alpha-amylase solution
- Lentil malt germinated for 120 h and mashed under a congress mashing regime (5D)
- Lentil malt germinated for 120 h and mashed under a congress mashing regime (5D-Ca) with the addition of calcium ions
- Lentil malt germinated for 120 h and mashed under a congress mashing regime (5D-Am) with the addition of alpha-amylase solution
- Lentil malt germinated for 144 h and mashed under a congress mashing regime (6D)
- Lentil malt germinated for 144 h and mashed under a congress mashing regime (6D-Ca) with the addition of calcium ions
- Lentil malt germinated for 144 h and mashed under a congress mashing regime (6D-Am) with the addition of alpha-amylase solution.

#### Lentil beer brewing

Mashing of the lentil malts for the lentil beer brewing was performed in the LB-12 mashing apparatus. Based on the results acquired during the congress mashing procedure, it was decided that the addition of alpha-amylase and an increase in the temperature during the final stage of the mashing is necessary. All lentil malts were used to produce beer, and Pilsener malt was used as the control sample. Each of the malts was mashed in duplicate. Mashing cups were filled with 200 cm^3^ of distilled water and heated to 45 °C. Fifty grams of ground malt samples were added to the mashing cups, and stirring (100 rpm) was started. After 30 min, the temperature was increased to 75 °C at a rate of 1 °C per min. After the temperature of the mash reached 75 °C, 100 cm^3^ of distilled water (75 °C) was added, followed by the addition of 0.01 cm^3^ of alpha-amylase solution (with the exception of Pilsener malt, to which alpha-amylase solution was not added). A temperature of 75 °C was maintained for 60 min. After 10 min, an iodine test was performed on all the mashes, and it turned out to be negative. After 60 min, the mashing cups were weighed, and the mass of the mashes was adjusted to 450 g using distilled water at 75 °C. Worts were then filtered, without cooling, through Macherey–Nagel paper filters. A total of 250 cm^3^ of clear wort was transferred to clean mashing cups, which were then inserted into the mashing machine and heated to 100 °C. Hop pellets were added to each of the cups (0.25 g) at the start of the boil. Wort was boiled for 60 min and then filtered through Macherey–Nagel paper filters into previously autoclaved 300 cm^3^ Erlenmeyer flasks, which were then cooled to 19 °C in an ice bath. Worts were inoculated with 1 cm^3^ *Saccharomyces cerevisiae* yeast solution and fermented in a cooled cabinet at 19 °C for 10 days. After fermentation, the beer was decanted from the yeast, degassed, mixed with diatomaceous earth (1 g per 100 cm^3^), filtered through a paper filter and collected for analyses. This beer brewing regime allowed us to obtain four different beer samples:

- Beer produced from the Pilsner malt sample (C)
- Beer produced from green lentil malt germinated for 96 h (4 days) (B4)
- Beer produced from green lentil malt germinated for 120 h (5 days) (B5)
- Beer produced from green lentil malt germinated for 144 h (6 days) (B6).

#### Analysis of the physicochemical parameters of lentil beers

Analysis of basic physicochemical parameters of the beer (alcohol content, extract content, density, real degree of attenuation, calorie content, beer color, and extract content of the wort) was conducted using a DMA 4500 Beer Analyzer (Anton Paar, Graz, Austria). Each beer was analyzed in duplicate, resulting in four readings per type of malt used.

#### Analysis of the volatile compounds present in lentil beers

The volatile profile of the beers was analyzed using the GC-FID method using a GC2010 Plus apparatus equipped with an FID-2010 detector and HS-20 headspace autosampler (Shimadzu Corporation, Kyoto, Japan). Volatiles were separated on a CP-WAX 57 CB capillary column (50 m × 0.32 mm ID × 0.2 µm) (Agilent Technologies, Santa Clara, CA, USA). Ten cm3 of degassed and filtered beer was transferred to 20 cm^3^ headspace vials. Each vial, before analysis, was conditioned and shaken in the headspace autosampler oven at 40 °C for 20 min. One cm3 of volatiles was withdrawn from the vial and transferred to the HS-20 headspace loop connected and injected onto the capillary column. The following oven temperature program was used for the separation of volatiles on the column: starting temp. : 40 °C (hold 3 min); temp. increase (5 °C/min) to 80 °C; hold for 3 min; temp. increase (10 °C/min) to 140 °C; hold for 9 min; temp. increase (20 °C/min) to 160 °C; hold for 4 min. Helium was used as the carrier gas, and the initial pressure of the gas was 100 kPa; the initial column flow was equal to 0.33 cm^3^/min; the initial linear velocity was equal to 11.8 cm/s; and the purge flow was set at 3 cm^3^/min. The FID was operated at 280 °C with a sampling rate of 40 ms. The flow of hydrogen gas to the FID was at a rate of 50 cm3/min, helium gas at a rate of 30 cm3/min and synthetic air at a rate of 400 cm3/min. Each produced beer was analyzed in triplicate (resulting in six repetitions per type of malt used)^18,19^.

#### Organoleptic analyses of the beer

Sensory analysis was performed by 5 trained panellists (4 men, 1 women, aged 28–34 years old). Beers were assessed by 10 point scale where 0 meant that the analysed attribute was absent and 9 indicated that the attribute was extremely strong. Parameters such as aroma (using 12 descriptors: fruity, alcoholic, citrusy, hoppy, DMS, grainy/cereal-like, malty, caramel, burnt/cooked, sulphuric, oxidised/aged, sweet) and taste (using 17 descriptors: fruity, alcoholic, citrusy, hoppy, DMS, grainy/cereal-like, malty, caramel, burnt/cooked, sulphuric, oxidised/aged, sweet, bitter, acidic, astringent, body, lingering) of the beverage were analysed^20^. Beers samples (10–15 cm^3^) were served in plastic clear cups in the sensory analysis rooms with red lights overhanging each of the panellist’s booths, which hid sample colour and transparency. Temperature of the served beer was 8 °C. The samples were coded with random three digit numbers. Panellists were not acquainted with the details of the tested samples.The organoleptic analyses and all methods connected with the sensory evaluation of the beverages produced in this study were carried out in accordance with relevant guidelines and regulations obligatory in the European Union, Poland and Wrocław University of Environmental and Life Sciences. All experimental protocols connected with the sensory evaluation were approved by The Polish Committee for Standardization and Bioethics Committee. Participants who performed sensory analysis, presented in the Supplementary Data, gave informed consent via the statement ‘I am aware that my responses are confidential, and I agree to participate in this survey’ where an affirmative reply was required to enter the survey. Panellists were able to withdraw from the survey at any time without giving a reason. The products tested were safe for consumption.

### Data analysis

Chromatographic data were integrated and quantified in LabSolutions software (Shimadzu Corporation, Kyoto, Japan). Identification of compounds was performed using analytical standards. Quantification was performed using external standards (described in Section "Reagents and standards".) based on a standard curve with five calibration points (coefficient of determination R2 was greater than or equal to 0.999). The results of the assessment of the technological parameters of the malts, physico-chemical parameters of the beers and composition of the volatiles in the beers were statistically analyzed in the Statistica 13 program from StatSoft (Tulsa, OK, USA) with one-way ANOVA (α = 0.05) using Tukey’s test. The results (as the means) of the organoleptic analysis are shown on the radar charts, constructed using Microsoft Excel 2010 (Microsoft, Redmont, WA, United States of America) software.

## Results and discussion

### Technological properties of the green lentil malts

The main goal of analyzing the technological properties of the malts is to assess whether the analyzed malt is characterized with parameters that would allow it to be used in the brewery to create wort. The best malts used in traditional beer brewing should yield a high volume of wort, characterized by a high extract content, quick saccharification time, short filtration time and pH in the range of 5.6 to 5.8^3^. The technological parameters of malts produced in this study are presented in Table 1 and were far from optimal but nevertheless better than those of all legume malts produced in previous studies^4,5^.

**Table 1:** Technological parameters of the lentil malts.

Sample^1^	Saccharification time (min)	Filtration time (min)	Wort volume (cm^3^)	Wort Extract (% w/w)	Wort pH	Wort viscosity (mPa/s)
4D	X	120 ± 0 a	175 ± 5 d	6.01 ± 0.02 a	6.01 ± 0.02 a	1.589 ± 0.035 a
4D-Ca	X	120 ± 0 a	180 ± 5 d	5.99 ± 0.02 ab	5.99 ± 0.01 ab	1.514 ± 0.024 ab
4D-Am	X	18 ± 2 c	240 ± 0 a	5.94 ± 0.03 bc	5.95 ± 0.01 c	1.484 ± 0.026 ab
5D	X	120 ± 0 a	185 ± 5 d	5.90 ± 0.04 d	6.00 ± 0.01 a	1.564 ± 0.004 a
5D-Ca	X	90 ± 1 b	212.5 ± 2.5 b	5.88 ± 0.03 d	5.89 ± 0.02 d	1.552 ± 0.033 a
5D-Am	X	17 ± 1 c	235 ± 5 a	5.95 ± 0.03 bc	5.95 ± 0.02 c	1.482 ± 0.019 ab
6D	X	120 ± 0 a	200 ± 0 c	5.95 ± 0.02 bc	5.95 ± 0.01 c	1.569 ± 0.011 a
6D-Ca	X	120 ± 0 a	200 ± 5 c	5.92 ± 0.03 cd	5.93 ± 0.02 cd	1.542 ± 0.029 ab
6D-Am	X	18 ± 1 c	235 ± 5 a	5.96 ± 0.03 abc	5.96 ± 0.01 bc	1.432 ± 0.014 b

^1^Values are expressed as the mean (n = 6 or n = 2 in the case of saccharification time and wort volume) ± standard deviation. Mean values with different letters (a, b, c, d) within the same column are significantly different (α = 0.05) according to Tukey’s test. Abbreviations are as follows: 4D—malt from green lentil germinated for 96 h, 4D-Ca—malt from green lentil germinated for 96 h mashed with the addition of calcium ions, (…), 6D-Am malt from green lentil germinated for 144 h mashed with the addition of alpha-amylase solution. ‘X’ stands for ‘no saccharification’ during mashing.

The main problem, which was recognized in malts produced in this study and was also seen in previous research, is the saccharification time of the malts. Typically, the addition of amylolytic enzymes should mitigate problems with slow saccharification time or the absence of saccharification, but all analyzed lentil malts had not saccharified during the congress mashing regime, despite the addition of amylolytic enzymes or calcium ions^21,22^. However, it is important to note that despite the lack of complete saccharification, the addition of calcium ions or amylolytic enzymes improved various parameters of the malt. Wort, after the congress mashing procedure, ought to filter during first 60 min to be described as ‘normal’ or ‘optimal’. Worts 4D, 5D, 6D, 4D-Ca and 6D-Ca were not filtered fully, and filtration was stopped after 120 min, according to the congress mashing procedure. Each malt type, when it was mashed with the addition of amylolytic enzymes (4D-Am, 5D-Am, 6D-Am), not only filtered fully but also filtered in a very short time, in the range of 17–18 min, over six times quicker than malts without the addition of enzymes. These data show that one of the most crucial factors impending lentil wort filtration is the presence of starch and various products of incomplete starch hydrolysis, such as dextrins, which have been previously proven to hinder wort and beer filtration^3,23^. The volume of the acquired wort was also the highest (equal to 235–240 cm^3^) in the samples with the addition of amylolytic enzymes, higher by 17.5–42.9% than the volume of the wort produced solely from the lentil malts. However, the addition of the amylolytic enzymes or calcium ions did not have a very significant effect on the extract content of the worts, decreasing it slightly (by 0.07% w/w) in sample 4D-Am or increasing it by 0.05% (w/w) in sample 5D-Am. The addition of calcium ions or amylolytic enzymes also resulted in worts with lower pH, but the decrease was in the range of 0.01–0.07 pH, and the resulting pH was closer to the recommended wort pH level in the 5.6–5.8^3^. The viscosities of the worts were similar, and the main difference could be seen in sample 6D-Am, which had the lowest viscosity of 1.432 mPa/s. The results acquired in this analysis not only show that green lentil malts lack an adequate amount of amylolytic enzymes to completely hydrolyze starch but also suggest that a temperature of 70 °C is too low to fully gelatinize all of the starch, which is why a higher temperature was used in the process of lentil beer brewing, and the data discussed in Section "Physicochemical parameters of the lentil beers" confirm this statement. In previous studies on malted lentils and other legumes, green lentil malts produced under the conditions typical for the production of Pilsner barley malt (45% moisture content of the germinating seed, five days of germination, temperature of germination equal to 15 °C) were characterized by very poor parameters in comparison to barley malt and all of the lentil malt samples produced in this study^4^. Worts acquired from the lentil malts in the 2021 study were characterized with extract content equal to 1.59% (w/w), which increased to 2.39–3.40% when they were mashed with the use of external amylolytic enzymes. These results show that significant improvement in the technological properties of lentil malt can be achieved by introducing simple changes to the steeping and germinating regime of the malting process and can possibly be improved to a greater extent in the future.

### Physicochemical parameters of the lentil beers

Beers brewed from the lentil malts were characterized with different basic physicochemical properties than the control sample, and the results of this analysis are shown in Table 2.

**Table 2:** Physicochemical parameters of the lentil beers.

Sample	Alcohol content (%v/v)	Beer extract content (%w/w)	Wort extract content (%w/w)	Density (g/cm^3^)	Degree of attenuation (%)	Energy content (kcal/100 cm^3^)	Beer color (EBC)
C	3.97 ± 0.01 a	3.07 ± 0.03 b	9.21 ± 0.01 a	1.00442 ± 0.00008 b	67.79 ± 0.04 a	32.72 ± 0.02 a	4.56 ± 0.07 c
B4	2.12 ± 0.09 b	3.70 ± 0.15 a	7.01 ± 0.13 b	1.00952 ± 0.00043 a	48.16 ± 1.23 b	24.82 ± 0.48 b	13.57 ± 0.10 a
B5	2.07 ± 0.03 b	3.88 ± 0.05 a	7.09 ± 0.04 b	1.01031 ± 0.00022 a	46.20 ± 0.19 b	25.13 ± 0.13 b	12.35 ± 0.57 b
B6	2.08 ± 0.04 b	3.68 ± 0.09 a	6.92 ± 0.05 b	1.00950 ± 0.00014 a	47.72 ± 0.92 b	24.47 ± 0.21 b	11.63 ± 0.45 b

^1^Values are expressed as the mean (n = 4) ± standard deviation. Mean values with different letters (a, b, c) within the same column are significantly different (α = 0.05) according to Tukey’s test. Abbreviations are as follows: B4—beer brewed from green lentil malt germinated for 96 h, B5—beer brewed from green lentil malt germinated for 120 h, B6—beer brewed from green lentil malt germinated for 144 h, C—beer brewed from standard Pilsner barley malt.

The starting extract content of the wort of the lentil beers was lower (6.92–7.09% w/w) than that in the C (9.21%), which most certainly influenced other parameters of the lentil beers, such as alcohol content, density and energy content. A higher extract content of the wort typically results in the production of beers with higher alcohol content because *Saccharomyces cerevisiae* yeast has more of the sugars to convert to ethanol^3^. This is certainly true in the case of the lentil beers brewed in this study, as the alcohol content of the B4, B5 and B6 samples is in the range of 2.07–2.12% v/v, compared to 3.97% in C. It is, however, important to note that the wort extract content of samples B4, B5 and B6 is lower by 30% than that in C, while the alcohol content of the lentil beers is lower by 48%. This shows that during the mashing processes, more of the extract transferred to the lentil wort comprised substances that cannot be fermented by the yeast. This statement is supported further by the degree of attenuation, which was lower by 20% in the lentil beers. This is not surprising, as the typical protein content of barley malt should not be higher than 11.5% of the dry weight, and lentil malts are characterized by a far higher protein content, up to 32.8%^3,5^. An experiment performed by Trummer et al.^6^ where 10% or 20% of barley malt was substituted by lentil malt, also resulted in worts with lower extract content. Differences in the density of the beers are because, first, B4, B5 and B6 are characterized by higher extract content in the finished beverage, and beer C is characterized by higher alcohol content, which, due to its low density, naturally decreases the density of the beer^3^. Various energy content of the analyzed beers is also rather simply explained, but it is not as straightforward to describe whether this trait is an advantage or disadvantage. The lower calorie content of the finished product is typically viewed as an advantage because obesity and obesity-related diseases are still increasing in developed countries^24^. This parameter could promote lentil beer over regular beer for various people who want to reduce their calorie intake, which could pave the road for the formulation of various novel beverages with reduced alcohol and energy contents. The last parameter, also differentiating lentil beers from the control sample, is the beer color. Beers produced from lentil malts were darker, characterized with color in the range of 11.63–13.57 on the EBC scale, with B6 and B5 being the lightest and B4 the darkest. Most of the compounds that influence the darker color of the malt are the result of the Maillard reaction^9^. In a recent study, Gasiński & Kawa-Rygielska^5^ showed that increasing the germination time for lentil malts reduces the concentration of starch, while the concentration of proteins remains similar. This would mean that there is lower amount of substrates in malts germinated longer (such as used for the brewing of B5 and B6) for the formation of Maillard products and results acquired in this study would confirm that statement. Analysis of the physicochemical parameters of the lentil beers has shown that there is a possibility of utilizing this type of material in the industry to produce fermented beverages; however, creating lentil beers characterized by the same properties as beers produced from barley malt can be problematic.

### Concentration of volatile compounds in the lentil beers

Currently, in the scientific literature, there are no data about the possible volatile composition of the beverage produced from fermented lentil malt wort. The sole scientific report about this topic can be found in the work by Trummer et al.^6^ where the aroma of the beer brewed with the addition of 10% or 20% lentil malt in the grain bill was described as ‘normal’, i.e., without ‘any unwanted odors’. GC-FID analysis allowed for the identification and quantification of 12 volatile compounds in the brewed beers (11 for sample C), as shown in Table 3, which are typically detected in fermented beverages^13,25^.

**Table 3:** Volatile compounds in lentil beers.

Compound	C^1^	B4	B5	B6
	(mg/dm^3^)			
Acetaldehyde	1.515 ± 0.356 d	3.574 ± 0.437 c	5.017 ± 0.580 b	7.675 ± 0.854 a
Ethyl acetate	7.332 ± 0.496 a	3.063 ± 0.714 b	3.272 ± 0.269 b	3.256 ± 0.224 b
2-butanol	n.d	0.377 ± 0.008 b	0.397 ± 0.009 a	0.395 ± 0.016 a
1-propanol	4.973 ± 0.375 b	2.816 ± 0.299 c	3.016 ± 0.048 c	13.959 ± 1.751 a
Isobutanol	6.343 ± 0.493 c	7.683 ± 0.168 b	8.438 ± 0.086 a	8.089 ± 0.261 a
Isopentyl acetate	0.489 ± 0.019 a	0.436 ± 0.002 b	0.437 ± 0.018 b	0.437 ± 0.014 b
2-methylbutanol	2.779 ± 0.222 a	1.532 ± 0.201 bc	1.505 ± 0.078 c	1.675 ± 0.123 b
3-methylbutanol	8.625 ± 0.700 a	5.559 ± 0.762 b	5.546 ± 0.450 b	6.368 ± 0.227 b
Ethyl hexanoate	0.061 ± 0.015 a	0.030 ± 0.003 b	0.026 ± 0.003 b	0.015 ± 0.001 c
Ethyl octanoate	1.374 ± 0.159 a	0.454 ± 0.011 b	0.484 ± 0.031 b	0.478 ± 0.021 b
Ethyl decanoate	2.534 ± 0.221 a	0.569 ± 0.346 b	0.379 ± 0.081 b	0.230 ± 0.193 b
Phenylethyl alcohol	3.446 ± 2.041 a	3.234 ± 1.208 a	3.628 ± 1.982 a	2.982 ± 0.996 a

^1^Values are expressed as the mean (n = 6) ± standard deviation. Mean values with different letters (a, b, c) within the same column are significantly different (α = 0.05) according to Tukey’s test. Abbreviations are as follows: B4—beer brewed from green lentil malt germinated for 96 h, B5—beer brewed from green lentil malt germinated for 120 h, B6—beer brewed from green lentil malt germinated for 144 h, C—beer brewed from standard Pilsner barley malt. N.d. stands for ‘not detected’.

Beers brewed from the lentil malts were characterized by higher concentrations of acetaldehyde, in the range of 3.574 ppm for B4 to 7.675 ppm for B6. Acetaldehyde is a volatile that is produced by the *Saccharomyces cerevisiae* yeast as a side-product of ethanol fermentation. In small concentrations, this compound can give beverages the aroma of green apples, but a greater concentration of this aldehyde significantly worsens flavor (etheric, pungent aroma)^26^. The concentration of acetaldehyde was below the threshold level in all the tested beers, but potentially, with the increase in the extract content (and, therefore, increase in pyruvate concentration, which is a substrate for the production of acetaldehyde) during the brewing of beers with increased extract content in the future, an increased concentration of acetaldehyde in the beverage might be troublesome for the production of lentil beers characterized by acceptable quality^13^. However, lentil beers were characterized by lower concentrations of various other components, which can create unwanted aromas in the volatilome of alcoholic beverages. One group of such components was higher alcohols, also called ‘fusel alcohols’. These volatiles have a pungent aroma described often as “solvent-like” and are produced by *Saccharomyces* yeast from amino acids via the so-called Ehrlich pathway^27^. During the production of alcoholic beverages, the presence of higher alcohols can be viewed as an advantage or disadvantage, depending on the desire of the producer. Very high concentrations of higher alcohols can be viewed as a defect in the production of spirits and lager beers, while high concentrations of particular alcohols (such as phenylethyl alcohol) are typical for wheat beers. However, higher alcohols play a crucial part in the formation of various esters, which are one of the largest and most important groups of compounds forming beer aroma, and lentil beers were characterized by significantly lower concentrations of these components^28^. Similarly, the ethyl acetate concentration, which imparts a fruity aroma to the beverages, was over two times higher in C than in B4, B5 or B6. This component was determined at a level below or on the verge of the detection limit in the lentil beers. The concentrations of ethyl hexanoate and ethyl octanoate were 2–4 times lower than those in C, although these components are characterized by very low odor threshold limits and were present in samples B4, B5 and B6 at detectable levels^29^. The concentration of ethyl decanoate was 5 times lower in B4 than in C, over 6.5 times lower in B5 and 11 times lower in B6. Formation of C6-C10 esters by the *Saccharomyces cerevisiae* yeast occurs through condensation reaction of ethanol and corresponding fatty acyl-CoA^30^. These results might indicate that the extraction of fatty acids from lentil malt is not as efficient as that from barley malt and that lentil reduces its concentration of C10 fatty acids during prolonged germination, but a more detailed analysis concentrating on the C6-C10 fatty acid content in lentil seeds/malts would be needed to confirm these hypotheses. Analysis of the volatile compounds in the lentil beers shows that the aroma of the produced beverage is not the same as that of the regular beer produced from barley malt but could possibly be adjusted by the brewers using various yeasts, fermentation temperatures and additives to acquire cereal-free beer with the typical, traditional aroma expected of this beverage.

### Aroma of the lentil beers

**Figure 1:**
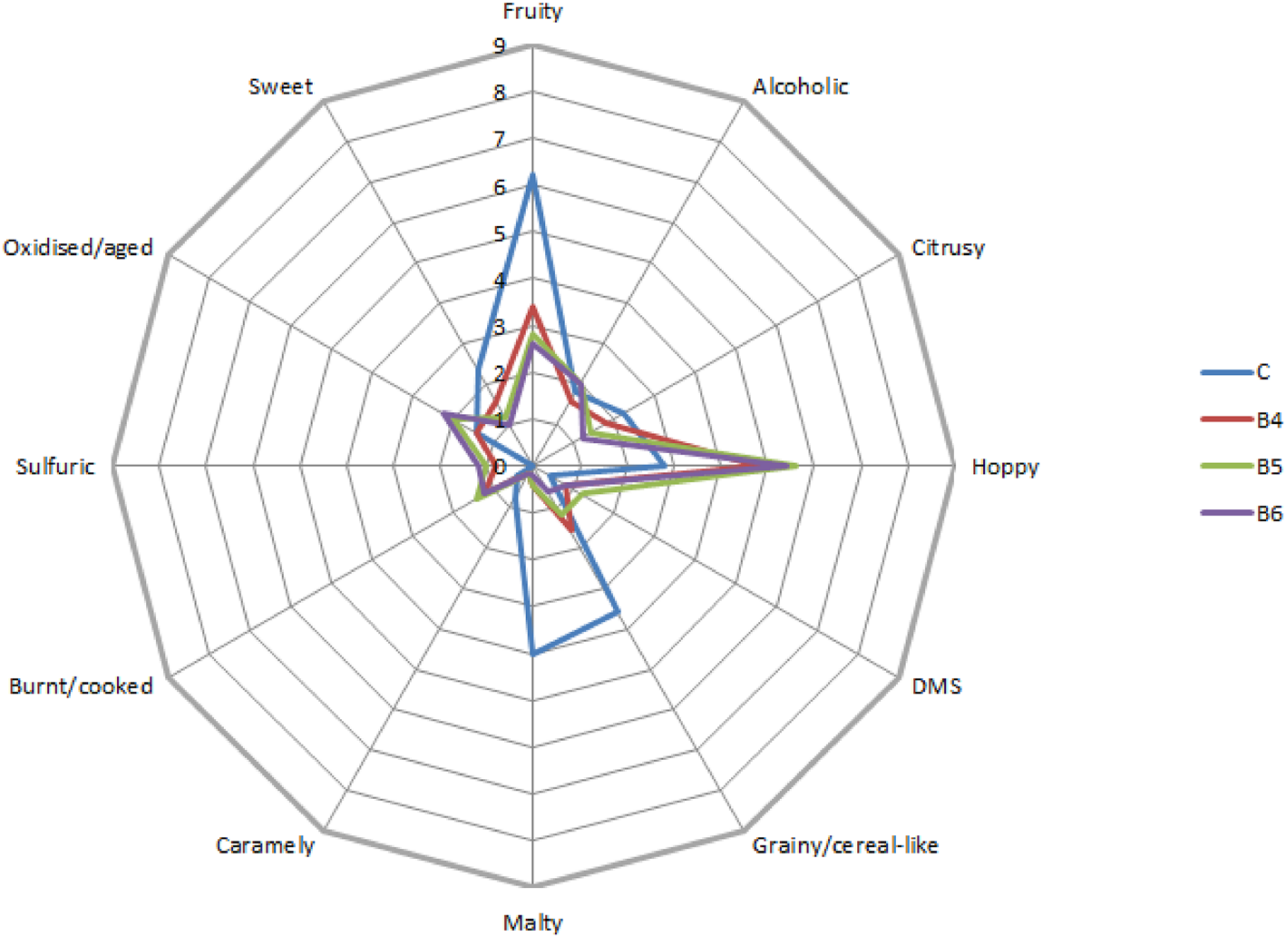
Radar chart showcasing results of the sensory analysis of the ‘aroma’ parameter of the lentil beers.

Results of the sensory analysis of ‘aroma’ parameter of the lentil beers are shown on Fig. 1.

Aroma of the beers produced from the lentil malt differed significantly from beer C in few aspects. C was characterised with aroma described as ‘fruity’ and possessed rather substantial ‘malty’ and ‘grainy’ notes, which were almost non-existent in the lentil beers. However, aroma of the lentil beers was characterised with more pronounced ‘hoppy’ notes. It is important to pinpoint that ‘citrusy’ aroma, which typically is more pronounced in highly-hopped beers was noted at very low level, in the C and B4, B5 and B6 alike^31,32^. It is furthermore important to note that lentil beers were not characterised with sulphur-like off-flavour, which is often associated with the legumes^33^. These results show that lentil malts could be used to produce gluten-free beers which would be characterised with rather neutral aroma.

### Taste of the lentil beers

**Figure 2:**
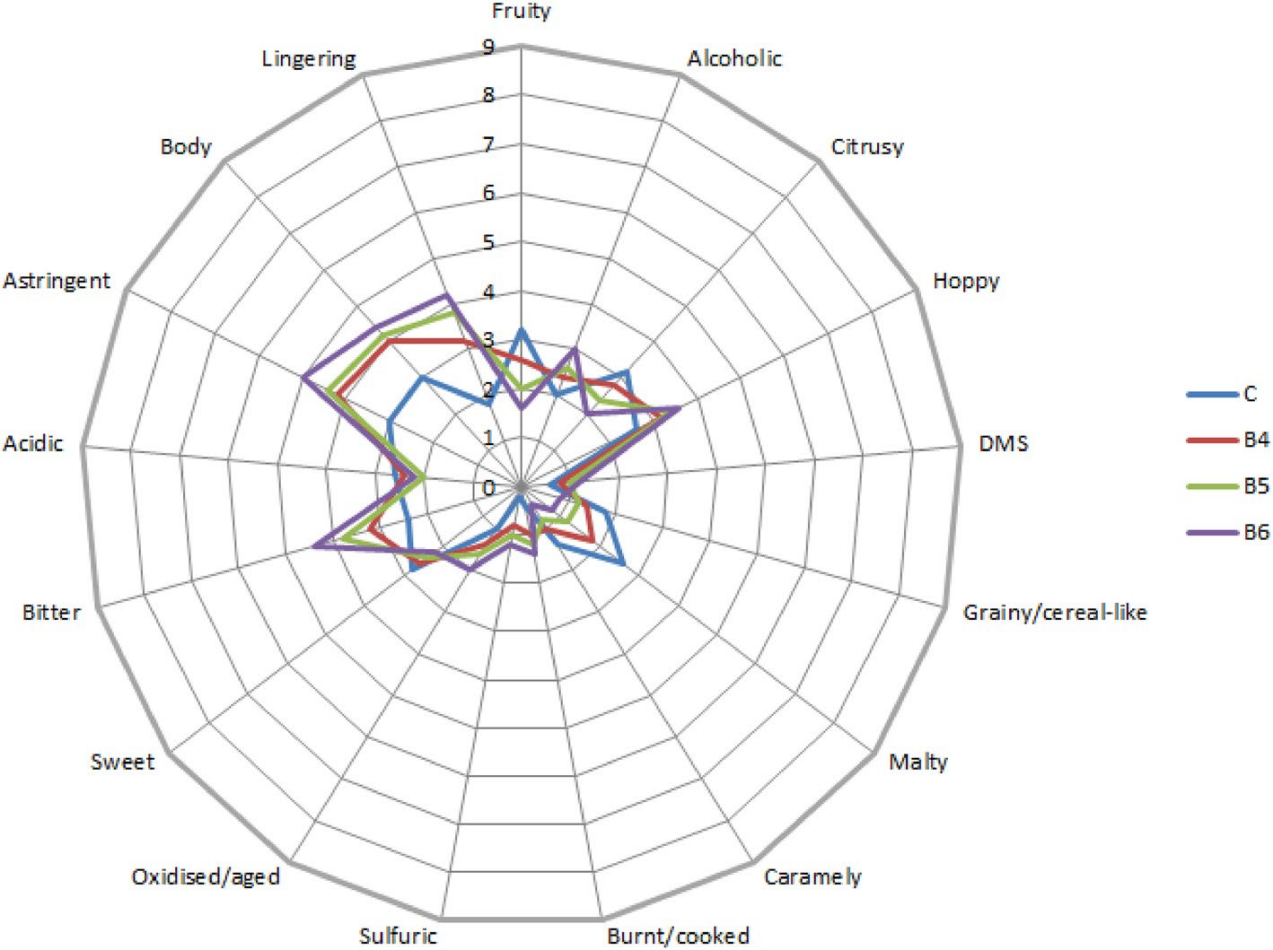
Radar chart showcasing results of the sensory analysis of the ‘taste’ parameter of the lentil beers.

Results of the sensory analysis of ‘taste’ parameter of the lentil beers are shown on Fig. 2.

Lentil beer taste was rated by panellists more negatively than the beer aroma. Lentil beers were characterised with more pronounced ‘lingering’ and ‘astringent’ taste than C. However, C was rated lower in the ‘body’ parameter, which is probably a result of the higher content of extract in the B4, B5 and B6. Panellists rated lentil beers as having slightly more pronounced ‘alcoholic’ taste than beer C. Additionally, as with the aroma, ‘hoppy’ taste was stronger in the lentil beers. This result might also be connected with the higher perceived bitterness of the lentil beers^34^. Taste of the C was rated higher only in parameters such as ‘fruity’, ‘citrusy’, ‘malty’ and by a very small discrepancy in the ‘sweet’ parameter, which corresponds well with the results of the ‘aroma’ analysis. It is however interesting to see that the C was not rated as less ‘sweet’ than the lentil beers, which were characterised with higher extract content.

## Conclusion

Data acquired in this study show that there is a possibility of producing cereal-free beers using only slightly changed methods used in the malting and brewing industry. Lentil malt produced by the method described in this manuscript can be successfully mashed with the use of only one type of external enzyme (α-amylase). However, the technology and methods of cereal-free beer brewing, using legume malts as substrates, are still very novel techniques, and beers produced in this way are characterized by some technological shortcomings. Beers produced from lentil malt are characterized by a lower degree of attenuation (which could be an advantage in the production of low-alcoholic beers) and lower calorie content than beers produced solely from barley malt. Additionally, various volatile components in the beer brewed from lentil malts are present in lentil beers in higher concentrations than in the beer brewed from barley malt, which could lead to various off-flavors in the perceived aroma. Results of the sensory analysis show, that the beers produced from the green lentil malt are not characterised with aroma or taste which is very distinct of the aroma of typical beer produced from barley malt. These results might indicate that there is a possibility of compiling recipes for the production of cereal-free beers adequate for people suffering from celiac disease or other gluten-related illnesses, which could be indistinguishable from typical beers popularly consumed throughout the world.

## Data Availability

The datasets used and analysed during the current study are available from the corresponding author on reasonable request.
