# Author Correction: Assessment of green lentil malt as a substrate for gluten-free beer brewing

**DOI:** 10.1038/s41598-024-53029-9

**Published:** 2024-01-30

**Authors:** Alan Gasiński, Joanna Kawa‑Rygielska

**Affiliations:** Department of Fermentation and Cereals Technology, Faculty of Biotechnology and Food Science, Wrocław University of Environmental and Life Science, Chełmońskiego 37 Street, 51-630 Wrocław, Poland

Correction to: *Scientific Reports* 10.1038/s41598-023-50724-x, published online 04 January 2024

The Funding section in the original version of this Article was incomplete.

"This work was supported by the Wroclaw University of Environmental and Life Sciences (Poland) as the Ph.D. research program "Innowacyjny Doktorat”, no. V, and the APC is financed by the Wroclaw University of Environmental and Life Sciences."

now reads:

“This work was supported by the Wroclaw University of Environmental and Life Sciences (Poland) as the Ph.D. research program "Innowacyjny Doktorat”, no. V. The article is part of a PhD dissertation titled 'Technological conditions in shaping the quality of special malts from legume seeds and the potential of their use in the food industry', prepared during Doctoral School at the Wrocław University of Environmental and Life Sciences. The APC is financed by Wrocław University of Environmental and Life Sciences.”

The original Article has been corrected.

